# A deconvolution method and its application in analyzing the cellular fractions in acute myeloid leukemia samples

**DOI:** 10.1186/s12864-020-06888-1

**Published:** 2020-09-23

**Authors:** Huamei Li, Amit Sharma, Wenglong Ming, Xiao Sun, Hongde Liu

**Affiliations:** 1 State Key Laboratory of Bioelectronics, School of Biological Science & Medical Engineering, Southeast University, Nanjing, 210096 China; 2 Department of Ophthalmology, University Hospital Bonn, 53127 Bonn, Germany

**Keywords:** Marker genes, Cellular fractions, Deconvolution, Acute myeloid leukemia, Subgroups, Diagnosis, Prognostic

## Abstract

**Background:**

The identification of cell type-specific genes (markers) is an essential step for the deconvolution of the cellular fractions, primarily, from the gene expression data of a bulk sample. However, the genes with significant changes identified by pair-wise comparisons cannot indeed represent the specificity of gene expression across multiple conditions. In addition, the knowledge about the identification of gene expression markers across multiple conditions is still paucity.

**Results:**

Herein, we developed a hybrid tool, LinDeconSeq, which consists of 1) identifying marker genes using specificity scoring and mutual linearity strategies across any number of cell types, and 2) predicting cellular fractions of bulk samples using weighted robust linear regression with the marker genes identified in the first stage. On multiple publicly available datasets, the marker genes identified by LinDeconSeq demonstrated better accuracy and reproducibility compared to MGFM and RNentropy. Among deconvolution methods, LinDeconSeq showed low average deviations (≤0.0958) and high average Pearson correlations (≥0.8792) between the predicted and actual fractions on the benchmark datasets. Importantly, the cellular fractions predicted by LinDeconSeq appear to be relevant in the diagnosis of acute myeloid leukemia (AML). The distinct cellular fractions in granulocyte-monocyte progenitor (GMP), lymphoid-primed multipotent progenitor (LMPP) and monocytes (MONO) were found to be closely associated with AML compared to the healthy samples. Moreover, the heterogeneity of cellular fractions in AML patients divided these patients into two subgroups, differing in both prognosis and mutation patterns. GMP fraction was the most pronounced between these two subgroups, particularly, in SubgroupA, which was strongly associated with the better AML prognosis and the younger population. Totally, the identification of marker genes by LinDeconSeq represents the improved feature for deconvolution. The data processing strategy with regard to the cellular fractions used in this study also showed potential for the diagnosis and prognosis of diseases.

**Conclusions:**

Taken together, we developed a freely-available and open-source tool LinDeconSeq (https://github.com/lihuamei/LinDeconSeq), which includes marker identification and deconvolution procedures. LinDeconSeq is comparable to other current methods in terms of accuracy when applied to benchmark datasets and has broad application in clinical outcome and disease-specific molecular mechanisms.

## Background

The orchestration of gene expression in appropriate spatiotemporal coordination helps to understand cellular function and differentiation, and how these processes are disrupted during the occurrence and development of diseases [1, 2]. In general, gene expression profiles are highly regulated and specific in different tissue/cell types, developmental stages, physiological conditions, external stimulations, and pathological conditions [2]. These specific genes, also termed as marker genes, can be used to determine the cell identity and help to understand the molecular mechanisms behind the diseases [3]. Especially for gene expression data of bulk samples, the marker genes act as a key to the accurate prediction of cellular fractions by the deconvolution algorithms. In recent years, a number of deconvolution methods (reference-based and reference-free) have been proposed to estimate the cellular fractions within the tissue samples [4–6], primarily, to gain better insight into the association between changes in cellular composition, disease situation and/or cellular development [7]. In most of the reference-based deconvolution approaches, the underlying marker genes of each cell type must be known in advance, and these marker genes are usually identified from the gene expression data of purified cell types. As Vallania et al. emphasize, the marker genes are the major determinant of deconvolution accuracy [8]. Therefore, a rational and effective approach to identify defined markers is crucial for the development of these deconvolution methods.

The marker genes basically are a subset of differentially expressed genes that provide unique information about individual cells. To date, most of the differential gene expression analysis methods including, DESeq2 [9], edgeR [10], limma [11] are based on the comparison of two conditions (cancer and control samples) with multiple biological repetitive experiments [2]. These approaches lack the possibility of obtaining cell type-specific marker genes that are expressed exclusively in one or two cell types and require comparison under multiple conditions. Although, few approaches such as RNentropy [2] and MGFM [3] were proposed to identify such markers across multiple conditions. RNentropy is an entropy-based tool that can detect markers of gene expression across multiple conditions using the log-likelihood ratio test [2]. While, MGFM selects markers based on the ratio of the second and first top expression value of a gene, and then assigns the markers to the cell type [3]. Nevertheless, as above mentioned, these existing methods have restrictions towards: (1), identifying a large number of low-expression markers as the marker genes; and (2), the membership (allocation) between the markers and cell types is rarely discussed.

Herein, we proposed a novel method to identify markers across multiple conditions on the basis of gene specificity score and mutual linearity strategies. The rationale behind using specificity scoring was mainly to select candidate marker genes, while the mutual linearity strategy was employed to allocate the selective candidate markers to cell types and to filter out the ones with low-confidence. Furthermore, the weighted robust linear regression (w-RLM) combined with the identified markers was applied to construct a reference-based deconvolution model. All these procedures mentioned above are packaged in our new tool, LinDeconSeq. Using this tool, we predicted the cellular fractions in patients of acute myeloid leukemia (AML) and then explored the possibility of clinical diagnosis.

## Results

### LinDeconSeq method

**Fig. 1:**
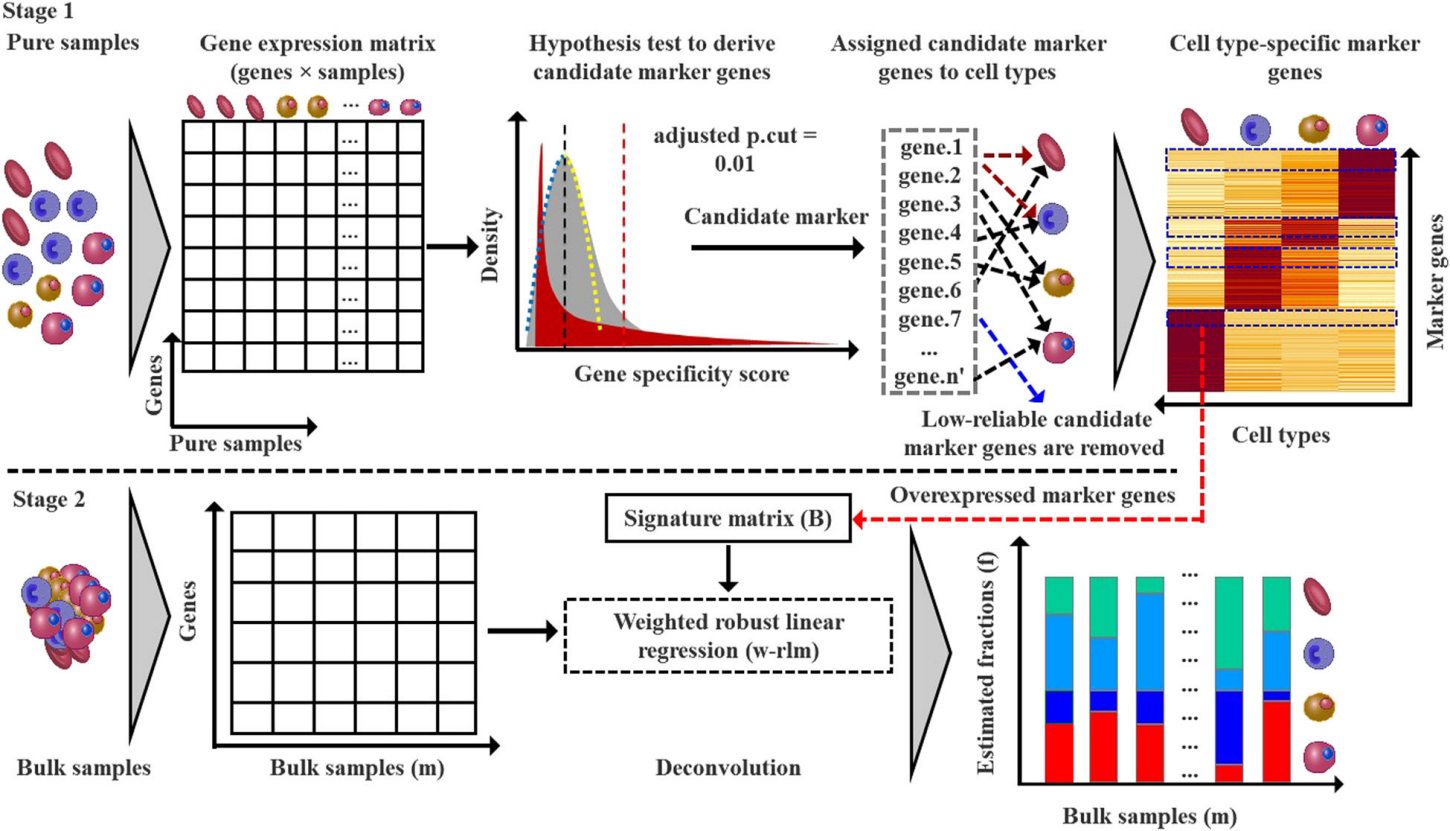
Flowchart of LinDeconSeq in identifying marker genes and predicting cellular fractions for bulk samples. The procedure has two stages. In stage 1, a set of marker genes are identified and allocated to the cell types. In stage 2, a weighted RLM with the signature matrix is derived from high confidence marker genes (identified in stage 1) and is used to predict the cellular fractions for bulk samples

In LinDeconSeq (Fig. 1, stage 1), the marker genes for each cell type were identified by integrating gene-specific scoring and mutual linearity strategy, mainly on the dataset of the purified cell populations. In this step, gene-specific scoring across heterogeneous cell types was a prerequisite for the subsequent marker screening. To measure gene specificity across different cell types in a robust and accurate manner, we first applied a method similar to Martı’nez et al. [12], which define the gene specificity and incorporate the weights of the genes transformed by the activation function (*tanh*) to ensure that the highly expressed genes are selected with greater probability (Methods section: Specificity scoring for each gene across all cell types). To avoid the condition where the candidate markers are arbitrary selected according to the rank of gene specificity scores, we generated the random specific scores by sampling and fitted the distribution with a normal distribution (Fig. S1A), which was further used to calculate the *P-values* and to determine the significance cutoff of candidate markers (Methods section: Selection of candidate marker genes by *z*-test). Secondly, since the marker genes of the same cell type were found to be highly correlated or mutual linearity (Fig. S1B), they were allocated to cell types using mutual linearity strategy (Methods section: Selection of seed markers and calculation of mutual linearity), in which the empirical *P-values* were produced by Monte Carlo sampling. The unassigned markers (*P-values* > 0.05) were considered unreliable and were removed out from the candidate marker gene set, the remaining ones were used as identification markers (Methods section: *P-value* estimation and allocation of candidate markers to cell types).

Since, the deconvolution of bulk samples is an important application scenario for the identification of marker genes, these deconvolution algorithms explicitly model the expression of a gene in a mixture as a linear combination of the expression within each cell type. Therefore, using previously identified high confidence marker genes (Methods section: Signature gene selection), weighted robust linear modeling (w-RLM) was integrated to predict cellular fractions for the bulk samples (Fig. 1, stage 2). To ensure that deconvolution remains accurate, robust and comprehensive, two major steps were considered: 1), only overexpressed marker genes of each cell type were chosen into the signature matrix (Methods section: Signature gene selection, Figs. S1C-E), and 2) used a weighted least squares approach [7] in combination with RLM to deconvolute bulk samples, which was more resilient to noise and eliminate the estimated fractions bias against each cell types (Methods section: Deconvolution).

Briefly, LinDeconSeq comprises procedures for both the identification of marker genes and deconvolution.

### Marker gene identification and deconvolution performance evaluation of LinDeconSeq on multiple datasets

In the dataset GSE74246 [13], which contains 49 fluorescence-activated cell sorting (FACS) purified RNA-Seq samples covering 13 primary human blood cell types, we identified 4558 out of 25,498 genes as marker genes using LinDeconSeq. Properly allocating candidate marker genes to cell types is an important issue for the cell identity and deconvolution (Methods section: Selection of candidate marker genes by *z*-test). Intuitively, if the cell types in the lineage are biologically close to each other, more of the marker genes would be shared by the cell types. In this work, we proposed a mutual linearity strategy to allocate the marker genes to cell types (Fig. 1, stage 1; Methods section: Selection of seed markers and calculation of mutual linearity). As expected, the higher Pearson correlation coefficient (PCC) between cell types, the more marker genes they share, such as between hematopoietic stem cell (HSC) and multipotent progenitor (MPP), and between CD4^+^ T and CD8^+^ T cells (Fig. S2A and B). For instance, gene *CD34,* which is a common marker for HSC, MPP and Lymphoid-primed multipotent progenitor (LMPP) in the CellMarker database (http://biocc.hrbmu.edu.cn/CellMarker/) [14], was correctly identified and allocated by LinDeconSeq. Likewise, few other genes such as *CD3D*, *CD2*, *CD5* and *CD7* were also correctly identified and allocated to CD4^+^ T and CD8^+^ T cells. These suggest that LinDeconSeq can accurately and reasonably find markers that are shared by different cell types.

**Fig. 2:**
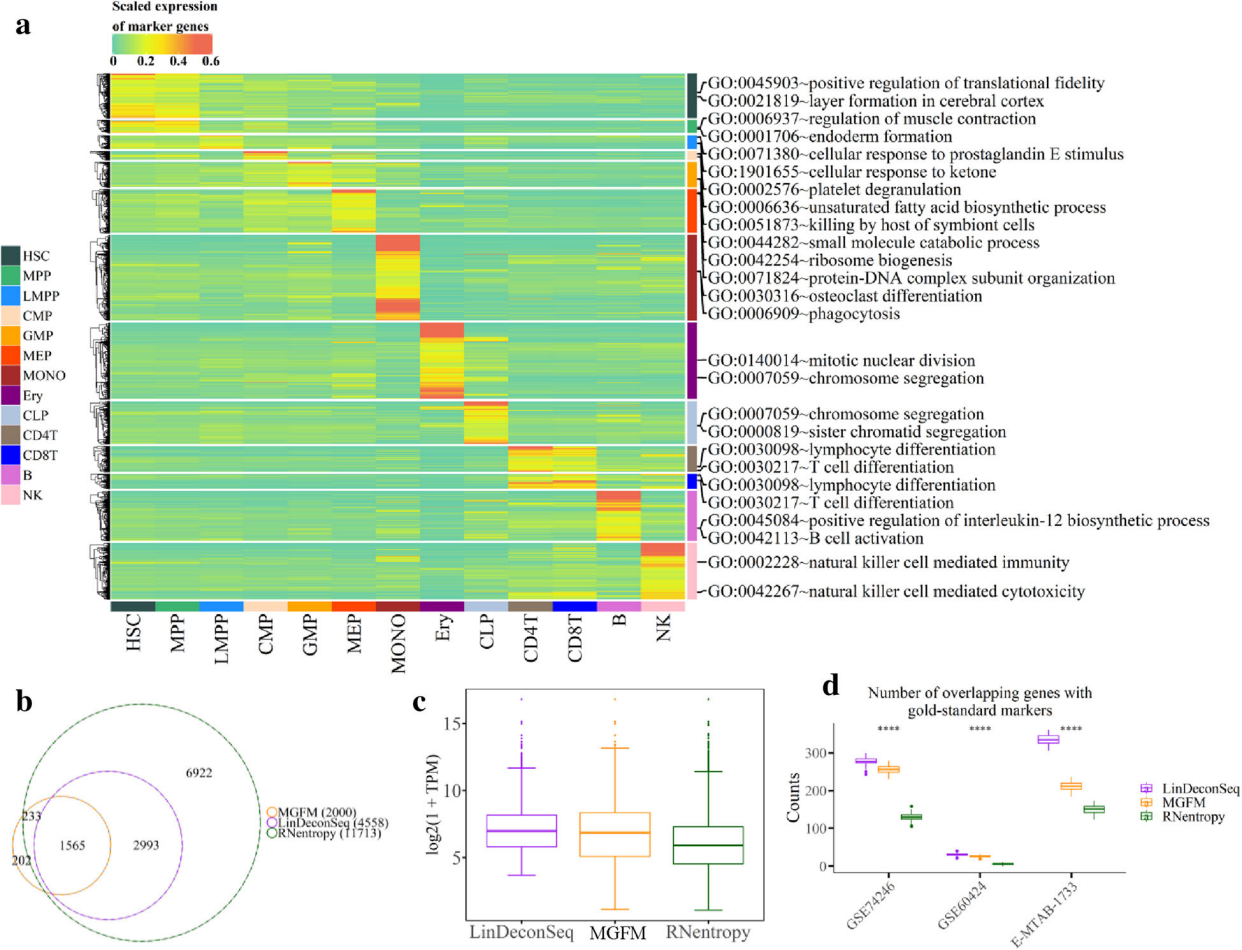
Evaluation of the reasonability of the identified marker genes. **a** Heatmap of expression of the marker genes. The expression was row-normalized (normalize each expression value by the sum over the row) across different cell types. The marker genes with the expression ≥0.6 are in red. Only the most biologically relevant two GO terms are shown for each module (Table S1). Hematopoietic stem cell (HSC), Multipotent progenitor (MPP), Lymphoid-primed multipotent progenitor (LMPP), Common Myeloid Progenitor (CMP), Granulocyte-monocyte progenitor (GMP), Megakaryocyte-erythrocyte progenitor (MEP), Monocytes (MONO), Erythroid progenitor (Ery), Common Lymphoid Progenitor (CLP), Natural killer cell (NK). **b** Venn diagram shows the overlapping of the marker genes by LinDeconSeq, MGFM and RNentropy on GSE74246 data set. **c** The distribution of the maximum expression of the identified marker genes among the given cell types. The thick line in the box represents the median value. The bottom and top of the boxes are the 25th and 75th percentiles (interquartile range). The whiskers encompass 1.5 times the interquartile range. **d** The distribution of the number of gold-standard marker genes in randomly chosen marker genes on the three datasets. The thick line in the box represents the median value. The bottom and top of the boxes are the 25th and 75th percentiles (interquartile range). The whiskers encompass 1.5 times the interquartile range. The statistical difference of the two groups was compared through the Wilcox test. *, *P < 0.05*; **, *P < 0.01*; ***, *P < 0.001*; ****, *P < 0.0001*

To illustrate that the marker genes are also able to accurately characterize the functional identity of the cell, an enrichment analysis was performed with GO terms. The enriched GO terms exactly represent the functional phenotype of each cell type (Fig. 2a, Table S1). For instance, the terms “natural killer cell (NK) mediated immunity and cytotoxicity” were enriched in NK cell type; “B cell activation” was enriched for B cell type; and the marker genes for CD4^+^ T and CD8^+^ T cells were enriched in “T cell and lymphocyte differentiation”. Hence, these results support the interpretability of the identification by LinDeconSeq.

To further test the reliability and accuracy of the marker genes by LinDeconSeq, we introduced RNentropy [2] and MGFM [3] for comparison. RNentropy is a recently proposed entropy-based method for the marker detection [2], while MGFM selects markers based on the ratio of second and first top expression value of the gene, which is incorporated into the CellFinder platform [3]. On the GSE74246 data set, the markers of LinDeconSeq were included in the maker gene list by RNentropy, while the markers of MGFM showed less overlap with those of LinDeconSeq (Fig. 2b). We noticed that a large number of genes (11713) were identified as the markers by RNentropy, perhaps by introducing some genes with lower expression levels. Figure 2c shows the gene expression distribution of the marker genes identified by three tools in the given cell types. LinDeconSeq has a higher median and a higher quartile (Fig. 2c), indicating that it has the potential to prevent false-positive markers of low-expression genes. We also compared the marker genes identified from the datasets GSE74246, GSE60424 [15] and E-MTAB-1733 [16] with the gold-standard marker list (Table S2). Briefly, for the GSE74246 dataset, we collected a total of 2060 markers from the CellMarker database as the gold-standard markers for 13 human primary blood cell types. Similarly, the GSE60424 dataset contains six immune cell types with 71 gold-standard markers, which were provided by the attachment of Amrani et al. [17]. While in case of E-MTAB-1733, similar information was described for ten human tissues and 2500 markers in the TiGER database [18] (Table S2). Since the number of markers identified by these tools varies widely (Figs. 2b, S2C and D), hence, to make the fair comparison, we applied non-replacement sampling to randomly select 1000 genes from the identified markers by each tool, and compared them with the corresponding gold-standard markers, this process was repeated for 100 iterations. Number of the overlapping genes are shown in Fig. 2d. LinDeconSeq also showed the highest median and highest average for the number of overlapping genes in each dataset, indicating lower false-positive detections and good performance (Fig. 2d). Here again, we randomly selected 60% of the markers from each gold-standard gene set and identified them using the three methods, and this step was repeated for 100 times. The comparisons showed that RNentropy has the highest median and highest average for the number of real markers, while the LinDeconSeq performs slightly worse (Fig. S2E). This can be explained as the *P-values* of genes calculated by RNentropy are over-significant, hence, the genes with low specificity in expression could not be filtered at this adjusted *P-value* threshold of 0.01.

As mentioned above, an important application scenario of the identified markers is for deconvolution, in which we first used the marker genes identified in the first stage to derive the signature matrix by minimizing the condition number (Methods section: Signature gene selection). Then the signature matrix and the w-RLM model were combined to predict the cellular fractions for bulk samples (Methods section: Deconvolution), and these processes were integrated into the LinDeconSeq. We compared LinDeconSeq with the other known three deconvolution methods: ls-fit [6], dtangle [5] and CIBERSORT [4]. Root-mean-square error (RMSD), Mean absolute deviation (mAD) and PCC was used to evaluate the performance of deconvolution on the published datasets from Liu’s [19], Shen-Orr’s [20] and Newman’s [4]. Briefly, Liu’s data set is an experimental data for a mixture of RNA-seq, (including HCC827 and NCI-H1975 cell types), Shen-Orr’s data consists of microarray analysis of rat liver, brain, and lung, while Newman’s data is based on blood samples from twenty adults of which the proportions of nine leukocytes types determined by flow cytometry (Table S0). All these datasets contain FACS-purified cell samples and bulk samples with known mixing proportions. Since with all these methods the preprocessing of the signature genes is required in advance, we first identified marker genes on these three datasets using LinDeconSeq and then extracted the overexpressed markers as signature genes. As shown in (Figs. S3A-C), the extracted signature genes were exclusively highly expressed in one cell type and weakly expressed in the others, indicating good specificity. As next, the selected signature genes were used to deconvolute the bulk samples with LinDeconSeq, ls-fit, dtangle and CIBERSORT, respectively. In the prediction of cell type fractions, LinDeconSeq showed lower RMSD and mAD between the predicted and real cellular fractions on the three data sets, indicating good robustness (Figs. S3D-G). Moreover, other deconvolution tools based on our identified signature genes also showed good prediction accuracy, which confirms the reliability of the marker genes identified by LinDeconSeq (Fig. S3G).

In summary, LinDeconSeq performed well in the comparisons in regard to marker genes identification and deconvolution.

### Cellular fractions have the potential to diagnose acute myeloid leukemia (AML)

**Fig. 3:**
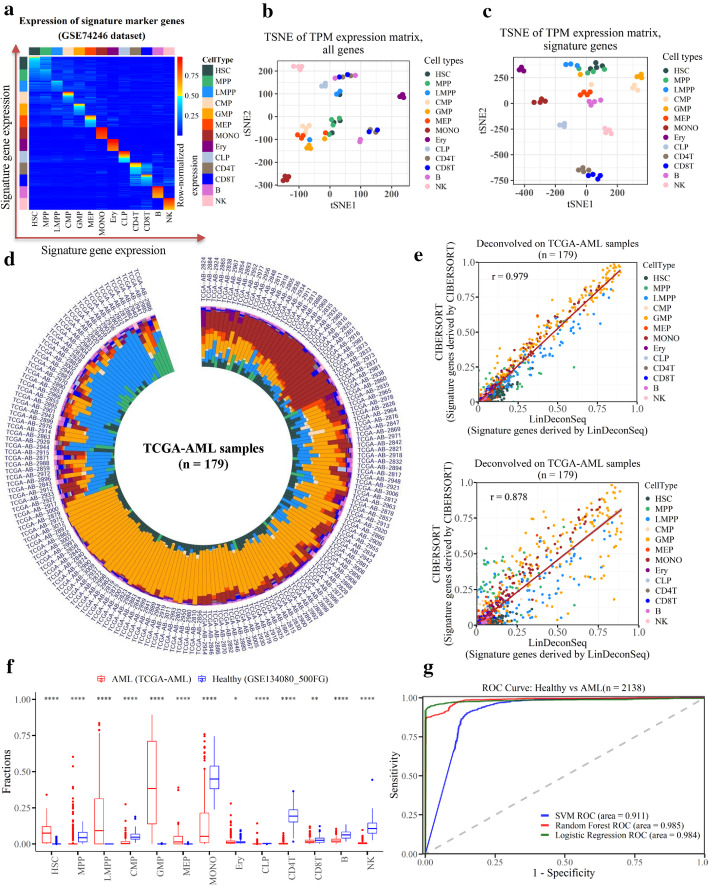
Deconvolution of TCGA-AML and healthy samples using LinDeconSeq. **a** Expression of the signature matrix. The expression was row-normalized. The upper bound of the color bar is 1. **b**-**c** t-SNE for all genes (**b**) and t-SNE for the signature genes (**c**), respectively. Each scatter represents a FACS-purified cell sample. **d** Circular bar plot of the cellular fractions for the 179 TCGA-AML patients. Each bar represents a sample and each color represents a specific cell type. The meanings of the colors are same as that in the legend of Fig. 2a. **e** The cellular fractions predicted by LinDeconSeq and CIBERSORT on the TCGA-AML patients. Each point represents a specific cell type in the sample. Pearson correlation coefficient (PCC, r) was calculated between the cellular fractions by LinDeconSeq and CIBERSORT. **f** The fractions of 13 primary blood cell types in AML and healthy samples. Within each group, each scatter represents the fraction of a specific cell type. The thick line in the box represents the median value. The bottom and top of the boxes are the 25th and 75th percentiles (interquartile range). The whiskers encompass 1.5 times the interquartile range. The statistical difference of the two groups was compared through the Wilcox test. *, *P < 0.05*; **, *P < 0.01*; ***, *P < 0.001*; ****, *P < 0.0001*. **g** ROC curve measuring the predictive based on the different cellular fractions between AML and healthy samples. The areas under (AUC) of ROC curve are 0.911, 0.985 and 0.984 for SVM, Random Forest and Logistic Regression, respectively

Following the deconvolution results, we investigated the differences in cellular fractions between AML samples and healthy samples. For this, we first used the marker genes of dataset GSE74246 [13] mentioned above to generate the signature matrix ***Ɓ*** with LinDeconSeq for subsequent deconvolution (Table S3). The signature gene exhibited over-expression in specific cell type, while it was low or unexpressed in others, indicating good specificity (Fig. 3a). Moreover, we used t-distribution stochastic neighbor embedding (t-SNE) [21] to visualize the samples with all the genes and the signature genes, respectively. The samples were better clustered with the signature genes than with all genes or with equivalent number (975) of the markers identified by RNentropy or MGFM, indicating good discrimination of cell types of the selected signature genes by LinDeconSeq, and thus providing a good basis for accurate deconvolution (Figs. 3b and c, S4A and B).

Secondly, we used LinDeconSeq with the signature matrix ***Ɓ*** to estimate the cellular fractions for 179 AML samples from the TCGA project (abbreviation: TCGA-AML) (Fig. 3d, Table S4) and 100 healthy samples (RNA-seq data of blood sample, GEO ID: GSE134080) (Fig. S5A and Table S5). In AML, the cell types GMP, LMPP and MONO dominated the components (Fig. 3d), but in healthy samples, the cellular fractions among these samples were quite similar (Fig. S5A), indicating that the cellular fractions differ between AML and healthy samples. In addition, AML is more heterogeneous in different individuals, while the components of different cell types in normal individuals show a favorable balance. We also compared LinDeconSeq to CIBERSORT, a tool that applies a two-sided unequal variance t-test by minimizing the condition number to obtain signature matrices, and then estimates the cell fractions using nu support vector regression (ν-SVR). We used the JAVA plugin of CIBERSORT [4] to generate a signature matrix and applied it to predict the cellular fractions on two datasets (179 TCGA-AML and 100 healthy). The results showed a high PCC and good concordance between LinDeconSeq and CIBERSORT when using the signature matrix derived from LinDeconSeq (Figs. 3e and S5B). When using their own signature matrices, both tools still show a good concordance (r > 0.87), but a lower PCC (Figs. 3e and S5B). These indicate that the cellular fractions predicted by LinDeconSeq are reliable. From a biological point of view, the four cell types (HSC, LMPP, MPP and GMP) dominated in TCGA-AML samples (Figs. 3d and f). This can be explained by the fact that these four cell types are closely related to three distinct stages of AML evolution, as also discussed previously [13]. For instance, the pre-leukemia HSC stage was most closely related to HSCs. Likewise, the leukemia stem cells stage exhibited strong similarity to GMPs and LMPPs, and leukemia blasts stage showed strong association with GMPs and MONOs. These results further show that the estimates of LinDeconSeq’s marker cell types of AML are consistent with the previous findings and the deconvolution of LinDeconSeq is reliable.

To further investigate the diagnostic value of cellular fractions for AML, we compared the cellular fractions of the healthy and AML samples in 13 cell types and found significant differences in most of them (Fig. 3f). Mainly, the cell types HSC, LMPP and Granulocyte-monocyte progenitor (GMP) showed significantly higher average fractions in AML, while the average fraction of MONO was significantly lower in AML. Importantly, HSC, LMPP and MONO remained closely related to the progression of AML and often serve as important markers in diagnosis, which is consistent with the previous findings [13]. In addition, we also compared the cellular fractions of male and female in TCGA-AML patients and observed no significant difference (Fig. S5C). We then used the median age of TCGA-AML patients to classify them into two categories, one above the median (older) and the other below or equal to the median (young). The results showed that GMP and NK cell types differ significantly between these two categories, with younger patients showing a higher GMP fraction (Fig. S5D). Overall, the cellular fractions can well characterize the differences between healthy and AML in comparison to gender, and can therefore be proposed as an early diagnostic marker of AML. Notably, the cellular fractions for specific cell types (GMP and NK) also showed significant differences in young and old patients.

Clinically, AML diagnosis is mainly based on chromosomal abnormalities [22]. However, in accordance with our analysis, the cellular fractions may provide a new perspective for the diagnosis. To this end, we constructed three diagnostic models to classify AML and healthy samples using the cellular fractions estimated by LinDeconSeq (Methods section: Cellular fractions estimation and classification for AML status). According to the predictions on the independent test datasets, these models include support vector machine (SVM), random forest and logistic regression with accuracy, precision, recall and F-score, all greater than 0.9. Importantly, the prediction performance of SVM was slightly lower than the other two (Fig. S5E). We further plotted the receiver operating characteristic (ROC) curves, the areas under curve (AUC) are 0.911 (SVM), 0.985 (Random Forest) and 0.984 (Logistic Regression), respectively (Fig. 3g). These indicate that the three classifiers constructed using the cell fractions are highly accurate in the diagnosis of AML diseases. The detailed predictions on the testing set (2138 samples) are shown in Table S6. To note, we trained the models with fewer samples (238 healthy and 583 AML samples) and tested them with additional samples (477 healthy and 1661 AML samples, Table S6). With an AUC of more than 0.90 on a data set of more than 2000 samples, our diagnostic model approach is reliable. Briefly, we demonstrated that the cellular fraction predicted by LinDeconSeq has the potential for early diagnosis of AML.

### Two biologically distinct subgroups of AML revealed on the basis of estimated cellular fractions

Using the estimated cellular fractions of the TCGA-AML samples, we calculated pairwise PCC of the cell type fraction between 13 cell types among the samples. The total number of pairs of cell types was 78 (13 × 12/2 = 78) and the PCCs of 28 pairs of these cell types were significant (t-test, *P-value* < 0.01) (Fig. S6A). The cell type GMP showed a negative correlation with MONO, LMPP, CD4T, B, CD8T, NK, MPP, and megakaryocyte-erythrocyte progenitor (MEP). The anti-correlation was also observed between MONO and LMPP, while GMP cellular fraction was positively correlated to HSC. Besides, LMPP, CD4T, B, CD8T, NK, MPP, and MEP showed mainly positive correlations with each other. These correlations depict a comprehensive landscape of cell lineages in AML progress, and suggest the potential value of AML subtyping.

**Fig. 4:**
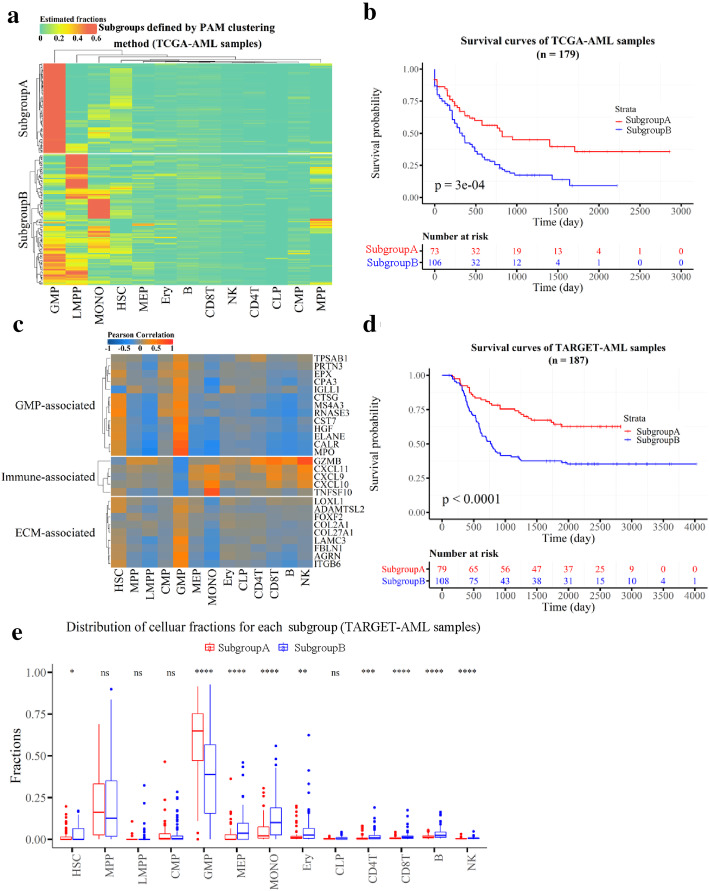
Two new AML subgroups revealed with the cell type fractions in the TCGA-AML data. Heatmap of the cell type fractions in the subgroups in TCGA-AML samples. **a** The subgroups are derived using PAM clustering method with the cell type fractions. Row represents TCGA-AML sample and column is cell type. **b** Kaplan–Meier curves for overall survival of the AML two subgroups for 179 TCGA-AML samples. *P-value* was from log-rank test. **c** PCCs between the cellular fraction and the expression of DEG across the 179 TCGA-AML samples. The subgroups show that the DEGs are associated with a particular function. **d** Kaplan–Meier curves for overall survival for the two subgroups that are predicted Random Forest classifier on the TARGET-AML data. The classifier is trained with the cell type fractions in TCGA-AML data. **e** The fractions of 13 primary blood cell types in the predicted subgroups for TARGET-AML samples. Within each subgroup, each scatter represents the fraction of a specific cell type. The statistical difference of the two groups was compared through the Wilcox test. *, *P < 0.05*; **, *P < 0.01*; ***, *P < 0.001*; ****, *P < 0.0001*

Given the complex of associations between different cell types, we performed PAM clustering based on the estimated cellular fractions to further define the subgroups (Methods section: Clustering for cellular fractions of TCGA-AML patients). We derived two clusters (SubgroupA and SubgroupB), which showed markedly different in the fractions of four cell types GMP, LMPP and MONO (Figs. 4a and S6B). The high fraction of GMP was the most striking feature of SubgroupA. The survival analysis also showed that SubgroupA has significantly better prognosis than SubgroupB (Fig. 4b, Table S7), which is consistent with the previously found that AML patients with higher GMP-like signals achieved significantly better outcomes [23]. In particular, it was found that the median age of SubgroupB was higher than that of SubgroupA with a significant difference (*P-value* < 0.01) (Fig. S6C). From a gender perspective, the percentage of males and females in SubgroupB was almost equal, while the proportion of females in SubgoupA was lower (Fig. S6D). To explore the underlying biological characteristics of the subgroups, enrichment analysis was performed on 967 differentially expressed genes (DEGs) (Table S8) between the two subgroups (Figs. S7A and B, Table S9). SubgroupA, which with a good prognosis, showed overexpression of the genes involved in extracellular matrix organization (ECM) and angiogenesis, while SubgroupB showed association with immune processes and correlation with poor prognosis (Figs. 4b and S7B).

Next, we investigated the PCCs between the cellular fractions and the expression profiles of the DEGs across the TCGA-AML patients. The results showed that the expression of the GMP’s marker genes such as *MPO*, *CALR*, *CST7*, *ELANE*, *HGF*, *EPX*, *MS4A3*, *CPA3*, *IGLL1*, *PRTN3*, *RNASE3*, *TPSAB1* and *CTSG* were highly correlated to the cell type fractions of GMP. Interestingly, the ECM-relevant transcripts of *LAMC3*, *COL27A1*, *COL2A1*, *FBLN1*, *ADAMTSL2*, *LOXL1*, *FOXF2*, *AGRN* and *ITGB6* were also highly correlated to the cellular fractions of GMP. In addition, immune-activated-related transcripts were highly correlated with the fractions of the immune cell types, including NK, B, CD8^+^ T and CD4^+^ T cells (Fig. 4c). The results suggest that the expression of the DEGs determines the phenotypic difference between the two subgroups, and this difference can be used to classify AML subgroups. To further verify this, we selected 214 genes from the DEGs by setting PCC ≥ 0.5 (between the expression and the cellular fraction) as features (Table S11) and performed a random forest classifier for the two subgroups on the TCGA-AML samples (accurate: 1, specificity: 1) (see Methods), which later on was tested on 187 TARGET-AML patients (Tables S0 and S12). The result again showed a significant difference in overall survival between these two subgroups, consistently to the observation in TCGA-AML samples (Figs. 4b and d). Furthermore, the cell type fractions in the two subgroups for the TARGET-AML samples also showed similar patterns with that of TCGA-AML samples (Figs. 4a and e, Fig. S6B, and Table S10), hence, GMP fraction appeared as the main difference between the two subgroups. Hence, the results indicate that the cell type fraction of GMP in blood can be considered as an important marker to classify the two subgroups and for the clinical prognosis (Notably, high GMP fraction indicates a good prognosis).

### Distinct mutations are associated with AML subgroups

**Fig. 5:**
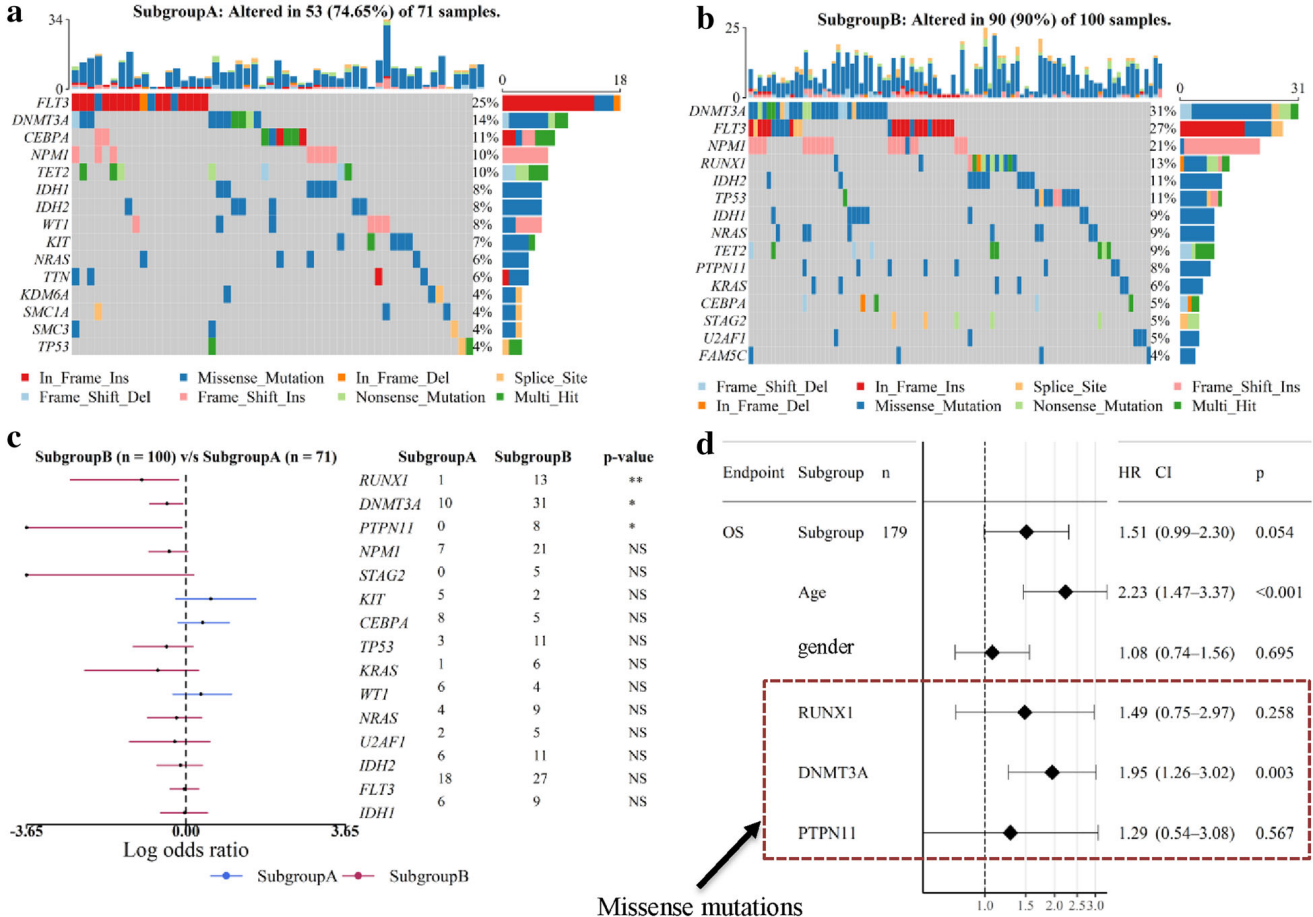
Mutation profile between the two subgroups derived from TCGA-AML data. **a**-**b** Mutation profile in the SubgroupA (**a**) and SubgroupB (**b**), respectively. Only top 15 genes are shown. **c** Forest plot of the differentially mutated genes between the two subgroups. Only genes with more than 4 mutations in the samples in one subgroup are included in analysis. The statistical difference of the two groups was compared through the Fisher exact test. *, *P < 0.05*; **, *P < 0.01*; ***, *P < 0.001*; ****, *P < 0.0001*. **d** Hazard ratios for overall survival associated with age (< 57 vs ≥57, median age = 57), subgroup (SubtypeA vs SubtypeB), gender and the number of missense mutations of *RUNX1*, *DNMT3A* and *PTPN11* in 179 TCGA-AML patients by multivariate Cox regression analysis. The length of the horizontal line represents the 95% confidence interval for each group. OS, Overall survival

We further investigated the mutations scenario for these two subgroups in TCGA-AML patients (Figs. 5a-c) and found that *KIT*, *CEBPA* and *NRAS* genes were frequently mutated in SubgroupA, while the mutations in *RUNX1*, *DNMT3A* and *PTPN11* genes were predominant in SubgroupB (*P ≤* 0.01, Fisher’s exact test). *RUNX1* encodes a sequence-specific transcription factor that is essential for HSC formation and for the differentiation of cells of lymphoid, myeloid and megakaryocytic lineages. Importantly, the mutations of *RUNX1* are known to be associated with the poorer prognosis of AML patients [24, 25]. Likewise, *DNMT3A* mutations are also highly recurrent in AML patients with an intermediate-risk cytogenetic profile, and are independently associated with a poor prognosis [26]. The protein encoded by *PTPN11* is an important signaling molecule that regulates a variety of cellular processes including cell growth, differentiation, mitotic cycle, and oncogenic transformation, and the mutations in this gene are also relevant towards AML [27, 28]. Hence, the presence of these mutated genes in our AML based analysis confirms the rationality of SubgroupB.

Next, we performed multivariate analysis that included age, established clinical risk (subgroup), gender and missense mutations of *RUNX1*, *DNMT3A* and *PTPN11*. The results show that the gender variable has a minor impact on the overall survival, established clinical risk (subgroup), age, while the missense mutations of *DNMT3A* play a major role in the progression and aggressiveness of AML (Fig. 5d). We further calculated the correlations between the cellular fractions and the mutation load across TCGA-AML samples. The analysis showed a negative correlation for the cell types HSC, GMP and MONO, while others/remaining cell types showed a positive correlation with the mutation load (Fig. S7C). Interestingly, the presence of GMP and HSC as the major cellular components in SubgroupA suggests that fewer mutations in these genes may be linked to a good prognosis, on the contrary, more/frequent mutation in LMPP are likely to be associated with a poor prognosis.

## Discussion

We presented here a hybrid tool, LinDecoSeq, which includes gene-specific scoring combined with mutual linearity strategy to identify marker genes across any number of cell types or other conditions. Besides, w-RLM approach is integrated by means of signature genes (overexpressed markers) derived from the first stage to predict cell type fractions of bulk samples. As compared to other available methods on multiple benchmark datasets, LinDeconSeq not only provides a quick and effective overview for the transcriptional variation among multiple cell types or other conditions, but also deconvolutes the cellular fractions in bulk samples to investigate the fundamental changes in cell state.

LinDeconSeq tries to catch the highly expressed and highly specific gene as marker gene. For this purpose, a factor was introduced which is transformed by activation function (*tanh*, Eq. 2) to balance the specificity and expression. LinDeconSeq also use mutual linearity strategy to map the candidate markers to the cell types and to further filter out the low confidence candidates. Given that our criteria is based on selecting the marker genes in highly expressed genes with filtered (twice cutoff) *P-value*, the number of identified marker genes in one or two cell types may be limited to few especially if the reference data contain many cell types.

Moreover, we used the cellular fractions predicted by LinDeconSeq to investigate the clinical diagnosis, subgroups and prognosis of AML. When comparing the cellular fractions between AML and healthy samples, we found AML associated distinct fractions in GMP, LMPP and MONO (Fig. 3f), which are known to be closely related to the onset and progression of AML [13, 29–31]. The differences in the fractions of human primary blood cell types between healthy and disease also showed potential application values for the early diagnosis of AML (Fig. 3g). Compared with the early diagnosis of AML based on gene expression profile [22], diagnosis based on cellular fractions provides a new perspective. Besides, the detailed analysis of differences in the cellular fractions in TCGA-AML patients revealed two distinct subgroups (SubgroupA and SubgroupB) for AML (Figs. 4a). GMP fraction was most pronounced between these two subgroups, particularly, in SubgroupA, which was also found to be strongly associated with the better prognosis. This conclusion was confirmed in TARGET-AML datasets (Figs. 4b and d), and was also consistent with the previous study that AML patients with higher GMP-like signals achieved significantly better outcomes [23]. The age distribution of TCGA-AML patients in these two subgroups exhibited significant differences, with the mean age of SubgroupB being higher and associated with a worse prognosis. This may be explained by the fact that AML is an aggressive hematological disorder that mainly affects elderly people [32]. The differentially expressed genes also showed a distinct and significant enrichment for the biological processes between SubgroupA and SubgroupB (Fig. S7B). While SubgroupA found to be closely associated with the extracellular matrix organization (ECM) and angiogenesis, for SubgroupB relates more to immune processes. The mutation analysis also showed differences between these two subgroups, as the frequent mutations in *RUNX1, DNMT3A* and *PTPN11* in SubgroupB were closely associated with a poor prognosis (Fig. 5c), which was consistent with previous studies [24–28]. It can therefore be speculated that the two subgroups of AML differ not only in clinical outcome but also in molecular mechanisms, which require further attention.

It is important to mention that the diagnosis analysis was purely based on the cellular fractions evaluated by LinDeconSeq. Whether the changes in cellular fractions can arise mainly caused by cancer or other intrinsic-extrinsic factors (virus infection or cell normal differentiation, development, medication/treatment response) remains to be investigated. In future studies, it will be equally important to define whether AML has any unique cellular fraction pattern. Besides, it is important to mention that clustering by t-SNE depends on some random initialization, thereby lack replicability of clustering results and include some artifacts, thus t-SNE data sets (Figs. 3b and c, S4A and B) requires additional steps of validation. Also, the estimation for the cellular fraction depends on the specificity of gene expression in reference samples, which can potentially vary since different construction strategies of the signature matrices.

## Conclusions

Taken together, we developed tool LinDeconSeq, which is freely-available and open-source, and includes procedures for the marker identification and deconvolution. When examining AML samples, we found that the cell type fractions evaluated by this tool can be used for the clinical diagnosis. Besides, we inferred the two new subgroups of AML which differ in both prognosis and mutation patterns.

## Methods

### Data normalization for gene expression datasets

For microarray datasets, the Robust Multiarray Averaging [33] (RMA) procedure was used for background correction and quantile normalization. For RNA-Seq datasets, gene expression profiles were normalized as the transcripts per million (TPM) quantification.

### Matrix of gene expression profiles of FACS-purified cell samples

The matrix was derived from the data of FACS-purified cell samples (Fig. 1, stage 1). The expression value was represented with TPM for RNA-Seq and with quantile normalization for the microarray data. The expression of replicated FACS-purified samples was averaged for each cell type, resulting in a *g* × *k* matrix **X**, where *g* is the number of genes and *k* the number of cell types.

### Specificity scoring for each gene across all cell types

The specificity score was calculated across *k* cell types for each gene in **X** using Eq. 1, which is similar to the gene specificity formula proposed by Martı’nez et al. [12].

1 $$ {\mathbf{S}}_i^{\hbox{'}}=\frac{1}{k}\cdot \sum \limits_{j=1}^k\left[\frac{{\mathbf{X}}_{ij}}{{\overline{\mathbf{X}}}_{i.}}\cdot {\log}_2\left(\frac{{\mathbf{X}}_{ij}}{{\overline{\mathbf{X}}}_{i.}}\right)\right] $$

Where **S**_*i*'_ is the specificity score, **X**_*ij*_ indicates the gene expression of the *i*th gene in *j*th cell type; X_i._ **X**_*i*._ is the expression of *i*th gene in each cell type, and ${\overline{\mathbf{X}}}_{i.}$ is the average value of **X**_*i*._; Notably, **S**_*i*'_ is near to zero when the expression of a gene is comparable in all cell types; and when the gene is exclusively expressed in a specific cell type, **S**_*i*'_ will be greater.

To ensure that the highly expressed genes are more likely to be chosen when the specificity scores are comparable, we added a *tanh*-transformed weight to the specificity score **S**_*i*'_ (Eq. 2).

2 $$ {\mathbf{S}}_i=\tanh \left(\lambda {\mathbf{W}}_i\right)\cdot {\mathbf{S}}_i^{\hbox{'}} $$

3 $$ {\mathbf{W}}_i=\frac{\max \left({\mathbf{X}}_{i.}\right)}{median\left\{\max \left({\mathbf{X}}_{t.}\right)|t=1,2,\dots, g\right\}} $$

Where **S**_*i*_ is the final specificity score for the *i*th gene; *λ* (0.1 as default) is a tuning parameter; and **W**_*i*_ is the weight for the *i*th gene. Since the gene expression is row-normalized based on the row-averaged expression in Eq. 1, the expression level of each gene is eliminated. **W**_*i*_ ensures that a gene with a higher expression can obtain a higher specificity score. For *λ*, since **W**_*i*_ ≥ 3, tanh(**W**_*i*_) barely changes and gradually converges to 1. This may lead to a smaller number of genes on which “tanh” has effects. Hence, the role of *λ* is to adjust the scope of “tanh” to the genes. The tanh(*λ***W**_*i*_) balances the expression level and specificity score by preventing a linear increase when the expression of a gene is incredibly high. For weakly expressed genes, the function (*tanh*) gives a low factor (the limit is 0), thus reducing the possibility that the gene is selected as a marker. For highly expressed genes, the *tanh*-transformed value is close to 1 so that the expression does not interfere with the contribution of specificity.

### Selection of candidate marker genes by *z*-test

To quantitatively determine the cutoff of *P-value* in the selection of candidate marker genes, we introduced a background distribution of the specific score by random sampling. Considering, the majority of genes from different cell types did not deviate much, we generated 100 random gene expression profiles for matrix **X** by sampling from a uniform distribution in the interval [min(**X**), max(**X**)] and then calculated the random specificity scores S^*^ for each gene using Eq. 2. According to the example of Fig. S1A, the distribution of S^*^ was close to a normal distribution and a large number of genes had specificity scores within or below the S^*^, it was consistent with our a priori hypothesis that most genes are not cell type-specific. Hence, in determining a cutoff for selecting markers, a normal distribution was fitted by using S^*^. As the model might be influenced by random outlier specificity scores, we first used the kernel density to find the center of the normal distribution by the “density” function (R, density(S*, method = gaussian)), and then fitted normal distribution under the center point using the “fitdistr” function (R package “fitdistrplus”) with the parameter “densfun = normal”. A *P-value* of each gene specificity score in **S** was determined by *z*-test based on the fitted normal distribution. The testing hypothesis of specificity score of gene *i* is Eq. 4.

4 $$ {H}_0={\mathbf{S}}_i\le {\mu}_{{\mathbf{S}}^{\ast }}\kern0.5em versus\kern0.5em {H}_1={\mathbf{S}}_i>{\mu}_{{\mathbf{S}}^{\ast }} $$

Where ${\mu}_{{\mathbf{S}}^{\ast }}$ is the mean of the fitted normal distribution and the genes with Benjamini–Hochberg [34] adjusted *P-value* ≤ 0.01 were considered as candidate marker genes.

### Selection of seed markers and calculation of mutual linearity

Ideally, the expression of cell type-specific gene is restricted to one cell type with robust expression across different biological replicates of the same cell type [35]. Thus, theoretically, if a candidate marker gene is exclusively expressed in an individual cell type, it is likely to be a marker of that particular cell type. Based on this fact, we employed **π**-value to measure the difference in gene expression of a specific cell type relative to the average expression of other cell types, and the **π**-value is defined by Eq. 5,

5 $$ {\boldsymbol{\uppi}}_i={\log}_2\left[\frac{\max \left({\mathbf{X}}_{i.}\right)}{\sum \limits_{j=1}^k{\mathbf{X}}_{ij}-\max \left({\mathbf{X}}_{i.}\right)}\cdot \left(k-1\right)\right] $$

The π _*i*_ will have a high value if a gene is exclusively expressed in a specific cell type, otherwise, it will be close to 0. Therefore, the candidate marker gene exclusively expressed in the *j*th cell type with the highest **π**-value was used as a seed marker gene for cell type *j*. This was performed for each cell type.

Due to the complexity of gene expression and close relationship between the cell lineages, it is difficult to allocate a candidate marker gene to specific cell types. For example, the marker gene of one cell type may be also overexpressed in some other cell types, which makes it obscure when mapping the gene to the cell type. Since marker genes belonging to the same cell type have similar expression patterns, they can be highly correlated or mutual linear (Fig. S1B). Therefore, we proposed a method based on the mutual linearity strategy combined with Monte Carlo sampling to map candidate markers to the cell types. And the linearity (**ρ**_*ij*_) between other marker gene and the seed marker gene was calculated for each cell type using Eq. 6,

6 $$ {\boldsymbol{\uprho}}_{ij}={\mathbf{r}}_{ij}^2\cdot \frac{1}{1+\exp \left(-{\boldsymbol{\uppi}}_i\right)}\cdot \operatorname{sgn}\left({\mathbf{r}}_{ij}\right) $$

Where **ρ**_*ij*_ is the degree of co-linearity between the *i*th gene and *j*th seed gene, **r**_*ij*_ is the Pearson Correlation coefficient (PCC) between the *i*th marker gene and the seed gene of cell type *j*. sgn(⋅) is a signum function. Since, the PCC between genes is affected by the degree of fluctuation (variance) of the expression data, the markers that may not be specific enough in themselves can also be highly correlated with the seed gene. To avoid this situation, the π value was introduced and integrated into Eq. 6.

### *P-value* estimation and allocation of candidate markers to cell types

To estimate the empirical *P-value* for each **ρ**_*ij*_ mentioned above, the Monte Carlo sampling was used, which allows us to test the null hypothesis, i.e. a candidate marker is indistinguishable from background genes. We first derived a null distribution **(**${\boldsymbol{\uprho}}_j^{\ast }$**)** for each cell type by calculating the linearity of the non-candidate marker genes and the seed marker genes using Eq. 6. And then ${\boldsymbol{\uprho}}_j^{\ast }$ was sorted in ascending order. Finally, the empirical *P-value* for each **ρ**_*ij*_ was estimated with Eq. 7.

7 $$ {\mathbf{P}}_{ij}=1-\frac{\min \left( which\left({\boldsymbol{\uprho}}_j^{\ast}\ge {\boldsymbol{\uprho}}_{i\mathrm{j}}\right)\right)+1}{number\kern1em of\kern1em background\kern0.4em markers\kern0.3em +1} $$

Where **P**_*i*j_ is a matrix, which row is for candidate marker gene and column is for cell type, each entry is the estimated *P-value* of **ρ**_*ij*._ As long as ${\mathrm{P}}_{{\mathrm{i}}^{\ast}\mathrm{j}}\le 0.05$ (default), we allocated the candidate marker gene *i* to the *j*th cell type. Otherwise, there is no difference between candidate marker gene *i* and background gene set and it was filtered out. The candidate markers were successfully allocated to the cell types as markers for the final identification of LinDeconSeq’s. These markers were stored in a list and recorded as **M** for subsequent selection of signature genes whenever deconvolution was required.

### Signature gene selection

Signature gene set is a subset of the marker gene sets (**M**). We sorted the markers of each cell type in M by descending order of **π** π-value, and then iteratively selected the top k marker genes (default range of k is from 50 to 200) of each cell type to generate signature matrices. The signature matrix (**B**) with the lowest condition number was retained (Fig. S1C). Notably, the condition number is an inherent matrix property, and the signature matrix with minimum value means that the linear system is more stable and can make deconvolution more accurate and robust (Figs. S1D and E) [6].

### Deconvolution

The gene expression profile of a bulk sample was considered as the convolution of the gene expression of the various cell types involved in the sample. Since, the main goal of deconvolution is to estimate the unknown cell type fractions based on the signature matrix [36], it can also be described with a linear regression, **m = f × B**, where **m** is the expression of bulk samples, **B** is the signature matrix and **f** is the coefficient indicating the changes in **m** with respect to **B**. Here, we performed the deconvolution using a robust linear model (RLM) that is more resilient to the noise. To further eliminate the estimated fractions bias against the cell types, we incorporated a weighted least squares approach previously described by Tsoucas et al. [7] into the RLM (w-RLM). The weighted least squares approach is capable to adjust the contribution of each gene in the optimal solution to mitigate bias due to the imbalances in gene expression levels. In other words, the contribution of a gene can be minimal if its average expression level is low. Hence, the approach has a positive effect on the elimination of prediction bias. When the deconvolution model converged, regression coefficients were extracted and negative regression coefficients were set to 0, then the remaining coefficients were normalized to sum to 1, yielding a vector representing the estimated cellular fractions.

When using LinDeconSeq to resolve the bulk samples, only three parameters need to be determined: bulks, signature matrix and weight (if weight is TRUE, w-RLM; Otherwise, RLM). For more details about the possible use, please visit: https://github.com/lihuamei/LinDeconSeq.

### Evaluation metrics

Given the actual cell type fractions **f** and the estimated fractions $\hat{\mathbf{f}}$, the deconvolution performance was evaluated by the following three metrics:

- Pearson correlation, PCC(***r***) = $Cor\left(\mathbf{f},\hat{\mathbf{f}}\right)$;
- Root mean squared error, RMSD = $\sqrt{avg{\left(\mathbf{f}-\hat{\mathbf{f}}\right)}^2}$;
- Mean absolute deviation, mAD = $avg\left(|\mathbf{f}-\hat{\mathbf{f}}|\right)$.

### Cellular fractions estimation and classification for AML status

We retrieved 49 FACS-purified RNA-Seq samples from GSE74246 [13], covering 13 primary blood cell types to derive signature matrix (***Ɓ***) (Supplementary Table S3), and further used them with the provided deconvolution tools to estimate the cellular fractions of bulk samples.

To verify whether the cellular fractions can be used to classify AML healthy status, we collected data of bone marrow and peripheral blood from 2959 individual samples (Tables S6). The cellular fractions of each sample were estimated with LinDeconSeq. To predict AML or healthy, 821 samples with estimated cellular fractions (including 238 healthy and 583 AML) were used to train Support vector machine (SVM) [37], Random Forest [38] and Logistic regression classification [39] models. The remaining 2138 samples with estimated cellular fractions (including 477 healthy and 1661 AML) were used as an independent test sets to validate the performance of the classifiers (Table S6).

### Clustering for cellular fractions of TCGA-AML patients

TCGA-AML samples with qualitatively different cellular fractions were prepared in advance. A partitioning around medoids (PAM) clustering was carried out for the prepared data using the “fpc” package with “asw” criterion using a Euclidean distance metric by the function “pamk”. The most robust number of clusters was then selected.

### Differentially expressed genes (DEGs) associating with the AML subgroups

DEGs between the AML subgroups were determined using the R package DESeq2 [9], and were further defined at the threshold of 2-fold change and Benjamini–Hochberg adjusted *P-value* ≤ 0.01.

### Functional enrichment analysis

Gene annotation enrichment analysis for the marker genes and DEGs was performed using the online tool DAVID (v6.8, https://david.ncifcrf.gov/) [40, 41]. Gene Ontology (GO) terms were considered statistically significant based on the Benjamini-Hochberg adjusted *P-value* < 0.05.

### Validation of the two prognosis-different subgroups using TARGET-AML patients

To validate the prognostic differences between the subgroups derived from TCGA-AML patients, we first calculated the PCCs between cellular fractions and the expression profiles of DEGs in 179 TCGA-AML patients. Genes with PCCs ≥0.5 were retained as features and their expression profiles were extracted to form a training set (Table S11). This training set with the subgroups derived from TCGA-AML patients was used to build a random forest classifier, which is based on default parameters provided by the R package randomForest [38]. Finally, we used the trained model to predict subgroups for TARGET-AML samples.

### Dataset

The data analyzed in this study are available from the Gene Expression Omnibus (accession numbers: GSE74246, GSE134080, GSE15061, GSE2842, GSE10258, GSE10358, GSE11375, GSE12417, GSE14468, GSE14479, GSE15434, GSE15932, GSE16028, GSE17114, GSE18123, GSE18781, GSE19743, GSE23025, GSE25414, GSE29883, GSE37642, GSE39088, GSE39363, GSE46449, GSE46819, GSE68833, GSE69565, GSE71226, GSE84334, GSE84844, GSE98793, GSE99039, GSE64098, GSE19830, GSE65133). The Cancer Genome Atlas Project and GDC Xena Hub (TARGET-AML gene expression and phenotype data sets were retrieved from https://xenabrowser.net/datapages/?cohort=GDC%20TARGET-AML&removeHub=https%3A%2F%2Fxena.treehouse.gi.ucsc.edu%3A443). The details about these data sets are summarized in supplementary Table S0.

## Supplementary information

**Additional file 1: Table S0.** Datasets and their application in this study. **Table S1.** Gene Ontology (Biological Processes (BP)) annotation for the marker genes identified by LinDeconSeq. **Table S2.** Gold-standard marker genes. **Table S3.** Signature matrix of 13 cell types revealed by LinDeconSeq in AML and Healthy samples. **Table S4.** The estimated cellular fractions by LinDeconSeq for 179 TCGA-AML samples. **Table S5.** The estimated cellular fractions for the 100 transcriptomic data of the healthy samples in GSE134080 using LinDeconSeq. **Table S6.** Comparison of the actual and predicted classification for 2138 independent test samples based on the SVM, Random Forest and Logistic Regression. **Table S7.** Two subgroups derived from TCGA-AML samples using PAM clustering method. **Table S8.** Differentially expressed genes (DEGs) revealed by DESeq2 between SubgroupA and SubgroupB. **Table S9.** Gene Ontology analysis for the DEGs with tool DAVID. **Table S10.** The estimated cellular fractions for the 187 TARGET-AML samples with LinDeconSeq. **Table S11.** The feature genes selected from the DEGs for training random forest classifier. **Table S12.** The subgroups predicted by the trained random forest classifier for TARGET-AML samples.

**Additional file 2: Figure S1.** Background to the model for marker genes selection and signature matrix generation. **Figure S2.** Evaluation of interpretability and accuracy of the marker genes identified by LinDeconSeq. **Figure S3.** Comparison of LinDeconSeq with the existing deconvolution methods on three benchmarking datasets. **Figure S4.** Clustering of the cells based on the marker genes determined by MGFM and RNentropy, respectively. Shown are *t-*SNE plots. **Figure S5.** Cellular fractions predicted by LinDeconSeq for healthy and TCGA-AML samples; And diagnostic performance of the different classifiers. **Figure S6.** Correlations between the cell type fractions and the characteristics of AML subgroups in age, gender and fractions. **Figure S7.** Differentially expressed genes between the AML subgroups; And the correlation between cell fraction and mutation load.

## Data Availability

All the scripts and methods proposed in this paper are available as an R-package at https://github.com/lihuamei/LinDeconSeq. All necessary arguments and information about samples to reproduce the results is present in Supplementary Tables.

## Abbreviations

AML: Acute myeloid leukemia
RMA: Robust Multiarray Averaging
TPM: Transcripts per million
RLM: Robust linear modeling
w-RLM: Weighted robust linear modeling
ROC: Receiver operating characteristic
AUC: Area under the curve
PCC: Pearson correlation coefficient
RMSD: Root-mean-square error
mAD: Mean absolute deviation
FACS: Fluorescence-activated cell sorting
DEG: Differentially expressed gene
GO: Gene Ontology
SVM: Support vector machine
t-SNE: T-distributed stochastic neighbor embedding
ECM: Extracellular matrix organization
HSC: Hematopoietic stem cell
MPP: Multipotent progenitor
LMPP: Lymphoid-primed multipotent progenitor
CMP: Common Myeloid Progenitor
GMP: Granulocyte-monocyte progenitor
MEP: Megakaryocyte-erythrocyte progenitor
MONO: Monocytes
Ery: Erythroid progenitor
CLP: Common Lymphoid Progenitor
NK: Natural killer cell
