# Supplementary material for: A deconvolution method and its application in analyzing the cellular fractions in acute myeloid leukemia samples
Source: BMC Genomics. 2020 Sep 23;21:652. doi: 10.1186/s12864-020-06888-1 (PMC7510109; doi:10.1186/s12864-020-06888-1)
Supplement: Supplementary file 2 — Additional file 2: Figure S1. Background to the model for marker genes selection and signature matrix generation. Figure S2. Evaluation of interpretability and accuracy of the marker genes identified by LinDeconSeq. Figure S3. Comparison of LinDeconSeq with the existing deconvolution methods on three benchmarking datasets. Figure S4. Clustering of the cells based on the marker genes determined by MGFM and RNentropy, respectively. Shown are t-SNE plots. Figure S5. Cellular fractions predicted by LinDeconSeq for healthy and TCGA-AML samples; And diagnostic performance of the different classifiers. Figure S6. Correlations between the cell type fractions and the characteristics of AML subgroups in age, gender and fractions. Figure S7. Differentially expressed genes between the AML subgroups; And the correlation between cell fraction and mutation load. [file 12864_2020_6888_MOESM2_ESM.pdf]

Supplementary Information

A deconvolution method and its application in analyzing the cellular fractions in Acute Myeloid Leukemia samples

Huamei Li[1], Amit Sharma[2], Wenlong Ming[1], Xiao Sun[1*], Hongde Liu[1]*

1, State Key Laboratory of Bioelectronics, School of Biological Science & Medical Engineering, Southeast University, Nanjing 210096, China;

2, Department of Ophthalmology, University Hospital Bonn, 53127 Bonn, Germany;

E-mails:

Huamei Li, li_hua_mei@163.com (HML);

Amit Sharma, Amit.Sharma@ukbonn.de (AS);

Wenlong Ming, 230189174@seu.edu.cn (WLM);

Xiao Sun, xsun@seu.edu.cn (XS);

Hongde Liu, liuhongde@seu.edu.cn (HDL).

*** Corresponding author:**

Xiao Sun, xsun@seu.edu.cn (XS)

Hongde Liu, liuhongde@seu.edu.cn (HDL)

1 Supplementary Figures

Figure legends

Figure S1 Background to the model for marker genes selection and signature matrix generation.

(A) Specificity score distribution. “Foreground” represents the specificity scores of genes from the true gene expression profiles (GSE74246); “Background” is the distribution of random specificity scores; “Fitted” is the normal distribution after fitting the random distribution (“Background”).

(B) Real marker genes expression patterns from B and NK cell types on the GSE74246 data set.

(C) Flow chart for signature matrix generation, which contains three steps. 1) Sorting marker genes of each cell type in M by π-value. 2) Iteratively selecting the top k marker genes of each cell type to generate signature matrices and calculate condition numbers. Default range of k is from 50 to 200. 3) The signature matrix that satisfies the minimum condition number can be chosen. Take 13 human primary blood cell types as an example.

(D) The relationship between condition number of signature matrix and RMSE (prediction vs. actual) on the basis of GSE19830 dataset.

(E) The relationship between condition number of signature matrix and correlation (prediction vs. actual) on the basis of GSE19830 dataset.

Figure S2 Evaluation of interpretability and accuracy of the marker genes identified by LinDeconSeq.

(A) Heatmap shows the pairwise PCC on the basis of the expression of allocated markers.

(B) Heatmap shows the marker index of each cell type. Marker index is the sum of the contributions of the markers allocated to the cell type. If the marker is uniquely allocated to a specific cell type, the contribution is 1, and if it is allocated to *j* cell types, the contribution is 1/*j*. The maker index greater than 100 appears red.

(C) Venn diagram shows the overlapping of the marker genes by LinDeconSeq, MGFM and RNentropy on GSE60424 data set.

(D) Venn diagram shows the overlapping of the marker genes by LinDeconSeq, MGFM and RNentropy on E-MTAB-1733 data set.

(E) The distribution of the number of identified marker genes which randomly chosen from gold-standard marker list. The thick line in the box represents the median value. The bottom and top of the boxes are the 25th and 75th percentiles (interquartile range). The whiskers encompass 1.5 times the interquartile range. The statistical difference of the two groups was compared through the Wilcox test. *, $P < 0.05$; **, $P < 0.01$; ***, $P < 0.001$; ****, $P < 0.0001$.

Figure S3 Comparison of LinDeconSeq with the existing deconvolution methods on three benchmarking datasets.

(A-C) Heatmaps of signature matrices derived from Newman’s (A), Liu’s (B) and Shen-Orr’s (C) datasets. The expression was row-normalized (normalize each expression value by the sum over the row), the upper bound of the color bar is 1.

(D-F) PCC heatmaps between the actual and predicted fractions by LinDeconSeq, CIBERSORT, dtangle and ls-fit on three benchmark datasets, respectively. (G) Newman’s, (H) Liu’s and (I) Shen-Orr’s.

(G) Table shows the average RMSD, mAD, and PCC (r) across all samples and cell types in Liu’s, Shen-Orr’s and Newman’s data set, respectively.

Figure S4 Clustering of the cells based on the marker genes determined by

MGFM and RNentropy, respectively. Shown are t-SNE plots.

(A-B) t-SNE for marker genes by MGFM and RNentropy, respectively. Each scatter represents a FACS-purified cell sample.

Figure S5 Cellular fractions predicted by LinDeconSeq for healthy and TCGA-AML samples; And diagnostic performance of the different classifiers.

(A) Circular bar plot of cellular fractions for 100 healthy RNASeq samples. Each bar represents a sample and each color represents a specific cell type.

(B) The cellular fractions predicted by LinDeconSeq and CIBERSORT on the healthy samples. Each point represents a specific cell type in the sample. Pearson correlation coefficient (PCC, r) was calculated between the cellular fractions by LinDeconSeq and CIBERSORT.

(C) The fractions of 13 primary blood cell types in male and female samples of TCGA-AML data. Within each group, each scatter represents fraction of a specific cell type. The thick line in the box represents the median value. The bottom and top of the boxes are the 25th and 75th percentiles (interquartile range). The whiskers encompass 1.5 times the interquartile range. The statistical difference of the two groups was compared through the Wilcox test. *, $P < 0.05$; **, $P < 0.01$; ***, $P < 0.001$; ****, $P < 0.0001$.

(D) The fractions of 13 primary blood cell types in younger (<57) and older (⩾57) samples of TCGA-AML data. Within each group, each scatter represents fraction of a specific cell type. The thick line in the box represents the median value. The bottom and top of the boxes are the 25th and 75th percentiles (interquartile range). The whiskers encompass 1.5 times the interquartile range. The statistical difference of the two groups was compared through the Wilcox test. *, $P < 0.05$; **, $P < 0.01$; ***, $P < 0.001$; ****, $P < 0.0001$.

(E) Performance measures of different classifiers.

Figure S6 Correlations between the cell type fractions and the characteristics of AML subgroups in age, gender and fractions.

(A) Correlation plot between the cell type fractions in 179 TCGA-AML samples. Blank boxes indicate non-significance at adjusted *P-value* > 0.01.

(B) The fractions of 13 primary blood cell types in the prediction of subtypes of TCGA-AML patients. Within each subtype, each scatter represents fraction of a specific cell type. The thick line in the box represents the median value. The bottom and top of the boxes are the 25th and 75th percentiles (interquartile range). The whiskers encompass 1.5 times the interquartile range. The statistical difference of the two groups was compared through the Wilcox test. **, P < 0.05; **, P < 0.01; ***, P < 0.001; ****, P < 0.0001.*

(C) Boxplot shows the age distribution of the two AML subgroups. Each scatter represents a specific sample from TCGA-AML data set. The thick line in the box represents the median value.

(D) Gender composition of each subgroup on TCGA-AML data set.

Figure S7 Differentially expressed genes between the AML subgroups; And the correlation between cell fraction and mutation load.

(A) The y-axis corresponds to the mean expression value of log10(adjusted *P-value*), and the x-axis displays the log2 fold change value. The red dots represent the up regulated expressed genes (adjusted *P-value* < 0.01, fold change ≥ 2) between SubtypeA and SubtypeB; the green dots represent the genes whose expression down regulated (adjusted *P-value* < 0.01, fold change ≤ -2).

(B) GO circle plot of significant enrichment biological process terms. The *zscore* calculated by $zscore = \frac{(up - down)}{\sqrt{count}}$, where up and down are the number of assigned

genes up-regulated in the SubtypeA or down-regulated, respectively.

(C) Pearson correlations between cellular fractions and mutation loads on TCGA-AML data set.

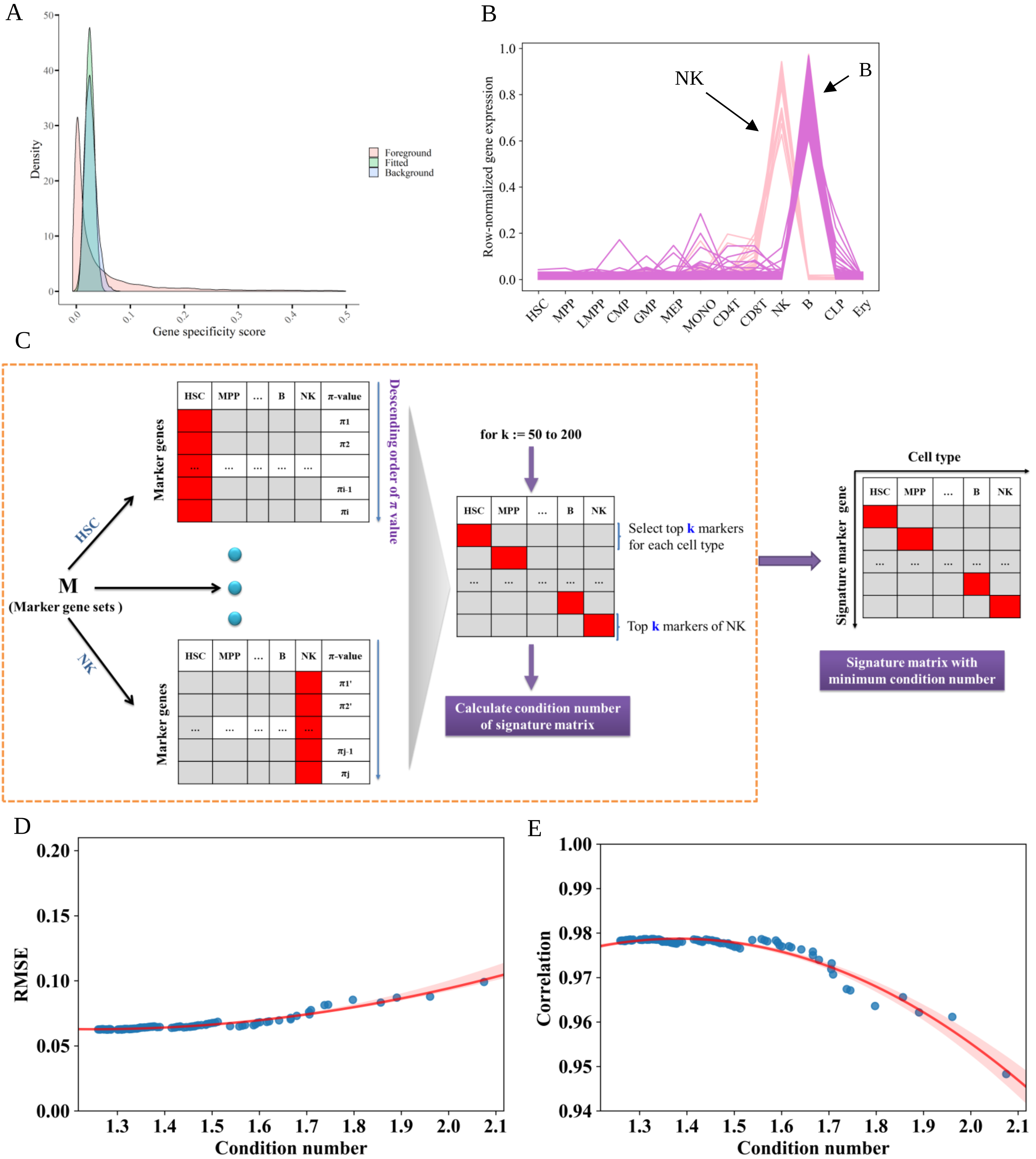
A
Density
Gene specificity score
Foreground
Fitted
Background
B
Row-normalized gene expression
NK
B
HSC
MPP
LMPP
CMP
GMP
MEP
MONO
CD4T
CD8T
NK
B
CLP
Ery
C
M
(Marker gene sets)
HSC
NK
Marker genes
π-value
Descending order of π value
for k := 50 to 200
Select top k markers for each cell type
Top k markers of NK
Calculate condition number of signature matrix
Cell type
Signature marker gene
Signature matrix with minimum condition number
D
RMSE
Condition number
E
Correlation
Condition number

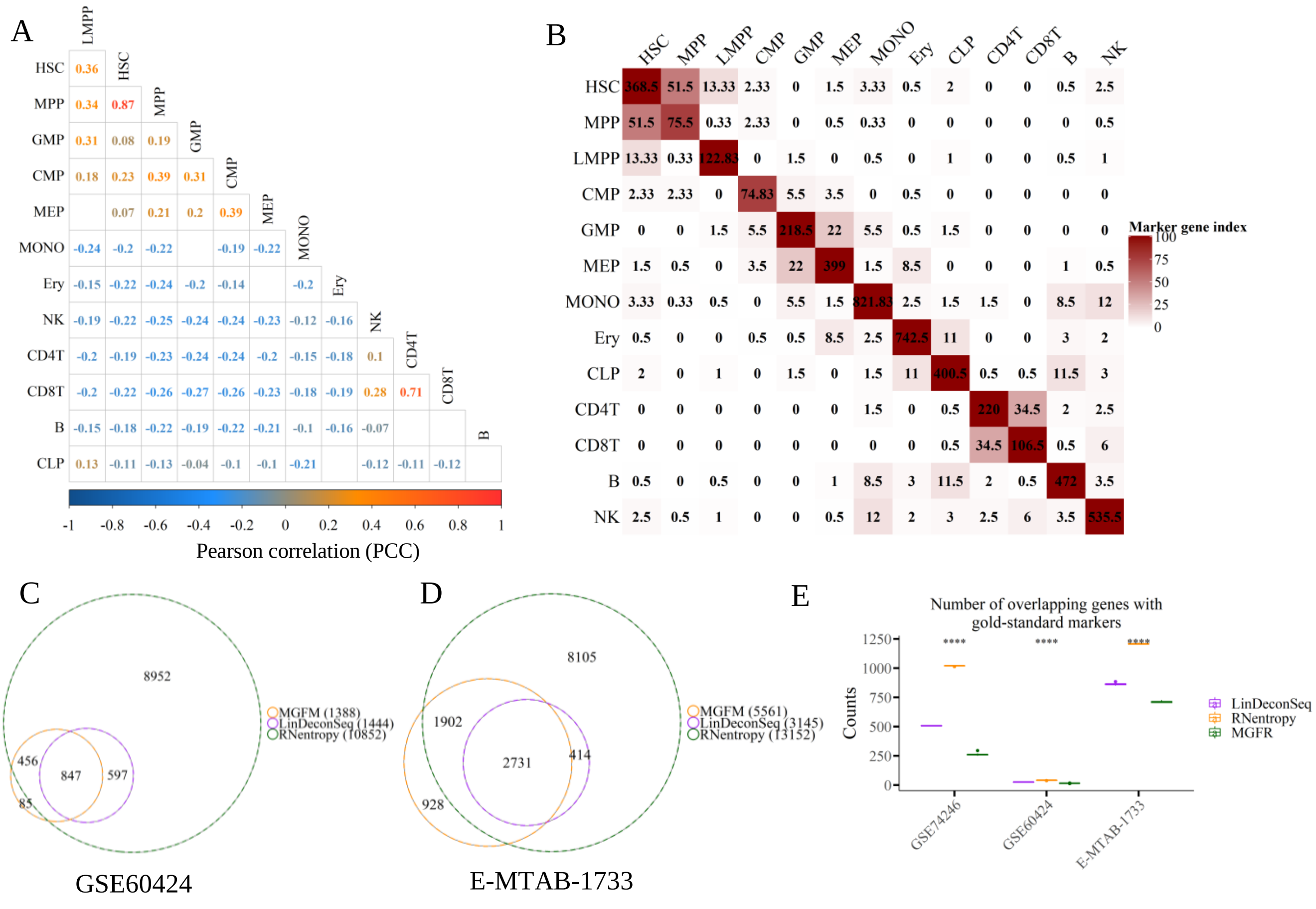

A
Pearson correlation (PCC)
B
Marker gene index
C
MGFM (1388)
LinDeconSeq (1444)
RNentropy (10852)
GSE60424
D
MGFM (5561)
LinDeconSeq (3145)
RNentropy (13152)
E-MTAB-1733
E
Number of overlapping genes with gold-standard markers
Counts
LinDeconSeq
RNentropy
MGFR
GSE74246
GSE60424
E-MTAB-1733

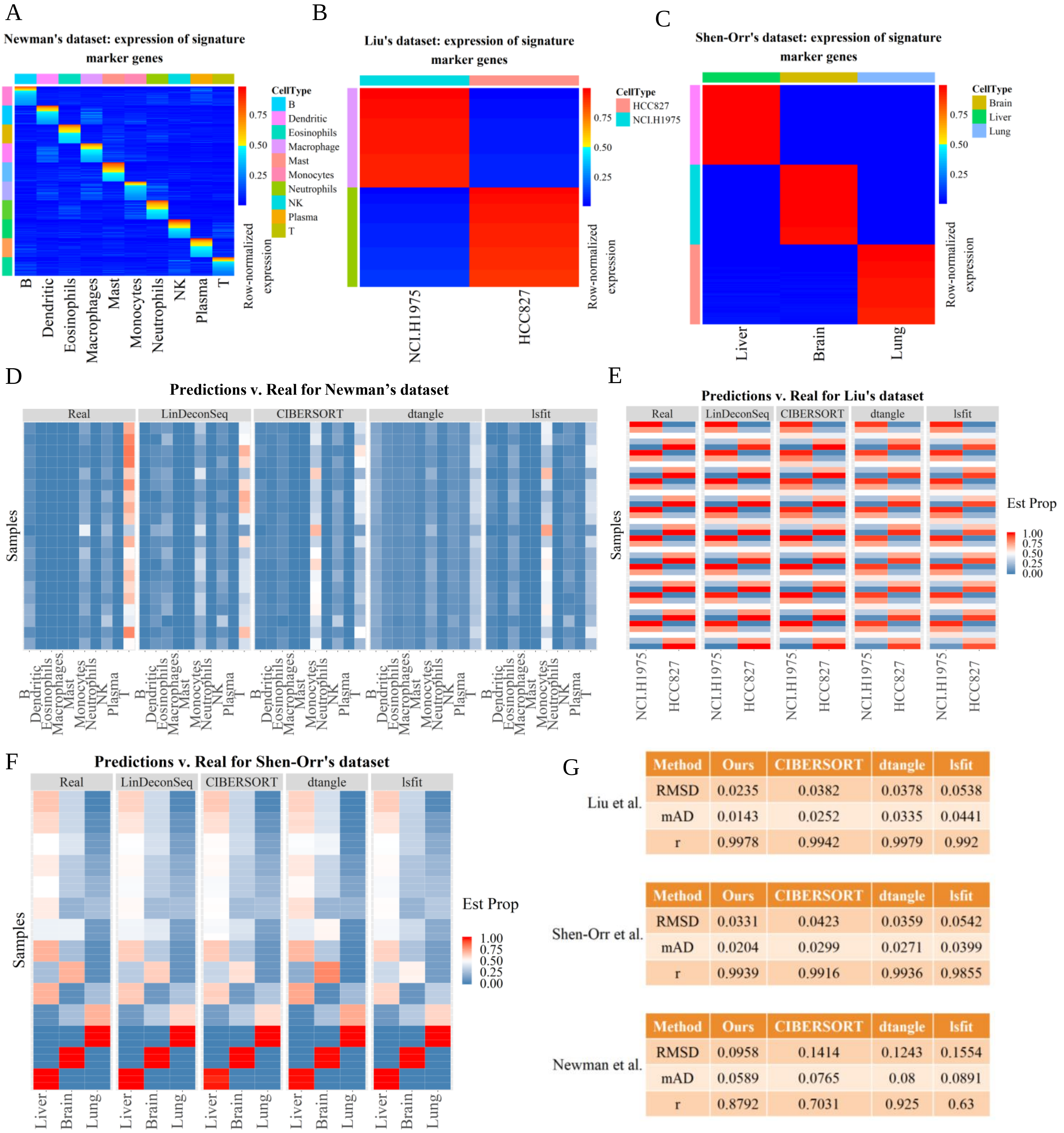

Liu et al.

Method	Ours	CIBERSORT	dtangle	lsfit
RMSD	0.0235	0.0382	0.0378	0.0538
mAD	0.0143	0.0252	0.0335	0.0441
r	0.9978	0.9942	0.9979	0.992

Shen-Orr et al.

Method	Ours	CIBERSORT	dtangle	lsfit
RMSD	0.0331	0.0423	0.0359	0.0542
mAD	0.0204	0.0299	0.0271	0.0399
r	0.9939	0.9916	0.9936	0.9855

Newman et al.

Method	Ours	CIBERSORT	dtangle	lsfit
RMSD	0.0958	0.1414	0.1243	0.1554
mAD	0.0589	0.0765	0.08	0.0891
r	0.8792	0.7031	0.925	0.63

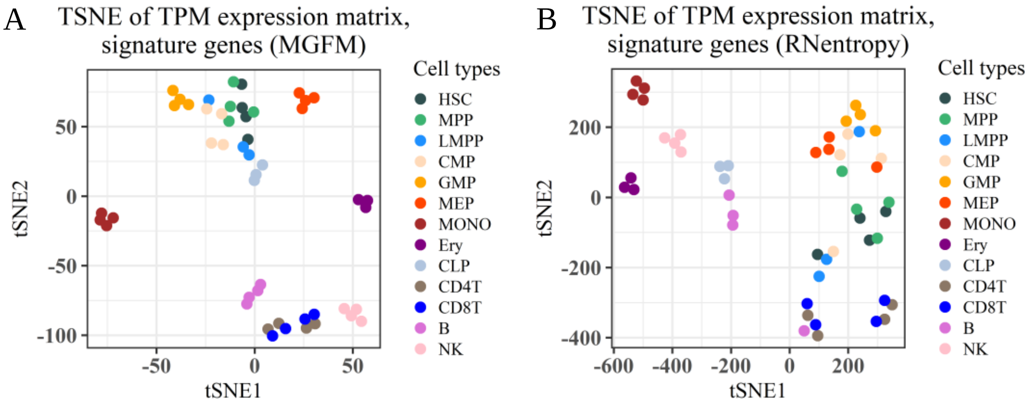
A
TSNE of TPM expression matrix, signature genes (MGFM)
tSNE2
tSNE1
50
0
-50
-100
-50
0
50
Cell types
HSC
MPP
LMPP
CMP
GMP
MEP
MONO
Ery
CLP
CD4T
CD8T
B
NK
B
TSNE of TPM expression matrix, signature genes (RNentropy)
tSNE2
tSNE1
200
0
-200
-400
-600
-400
-200
0
200
Cell types
HSC
MPP
LMPP
CMP
GMP
MEP
MONO
Ery
CLP
CD4T
CD8T
B
NK

A

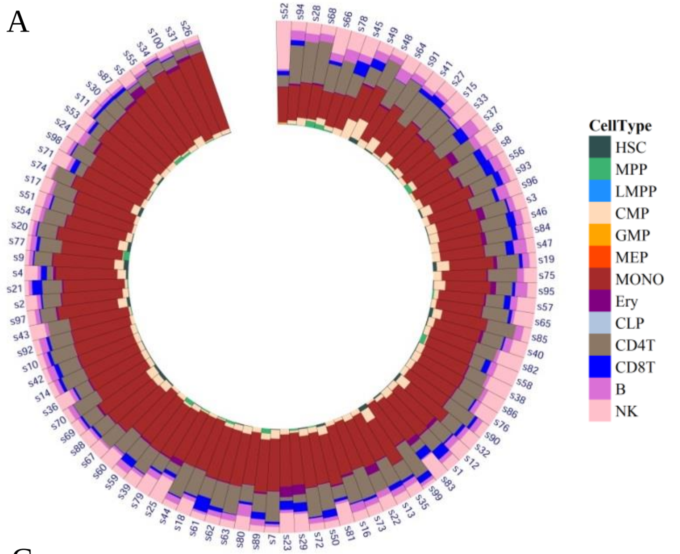

B

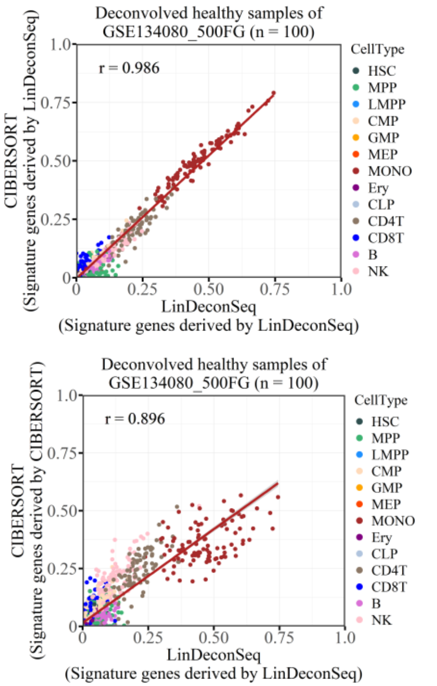

C

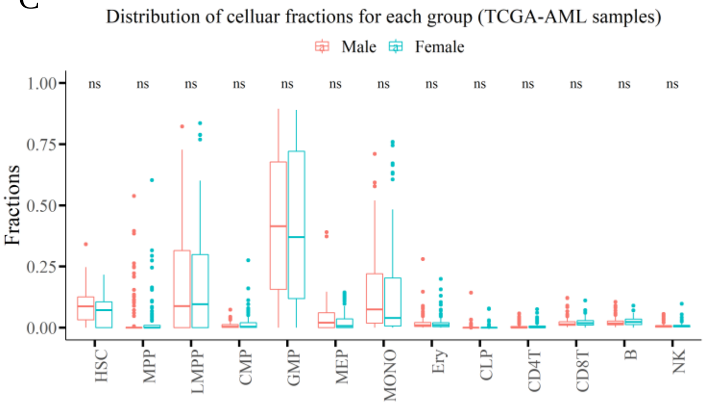

D

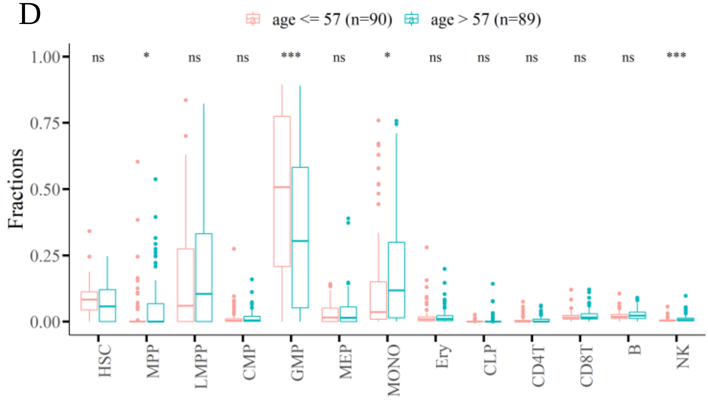

E

Classifier	Accuracy	Precision	Recall	F-Score
SVM	0.916	0.928	0.966	0.947
Random Forest	0.944	0.943	0.988	0.965
Logisitic Regression	0.956	0.989	0.954	0.971

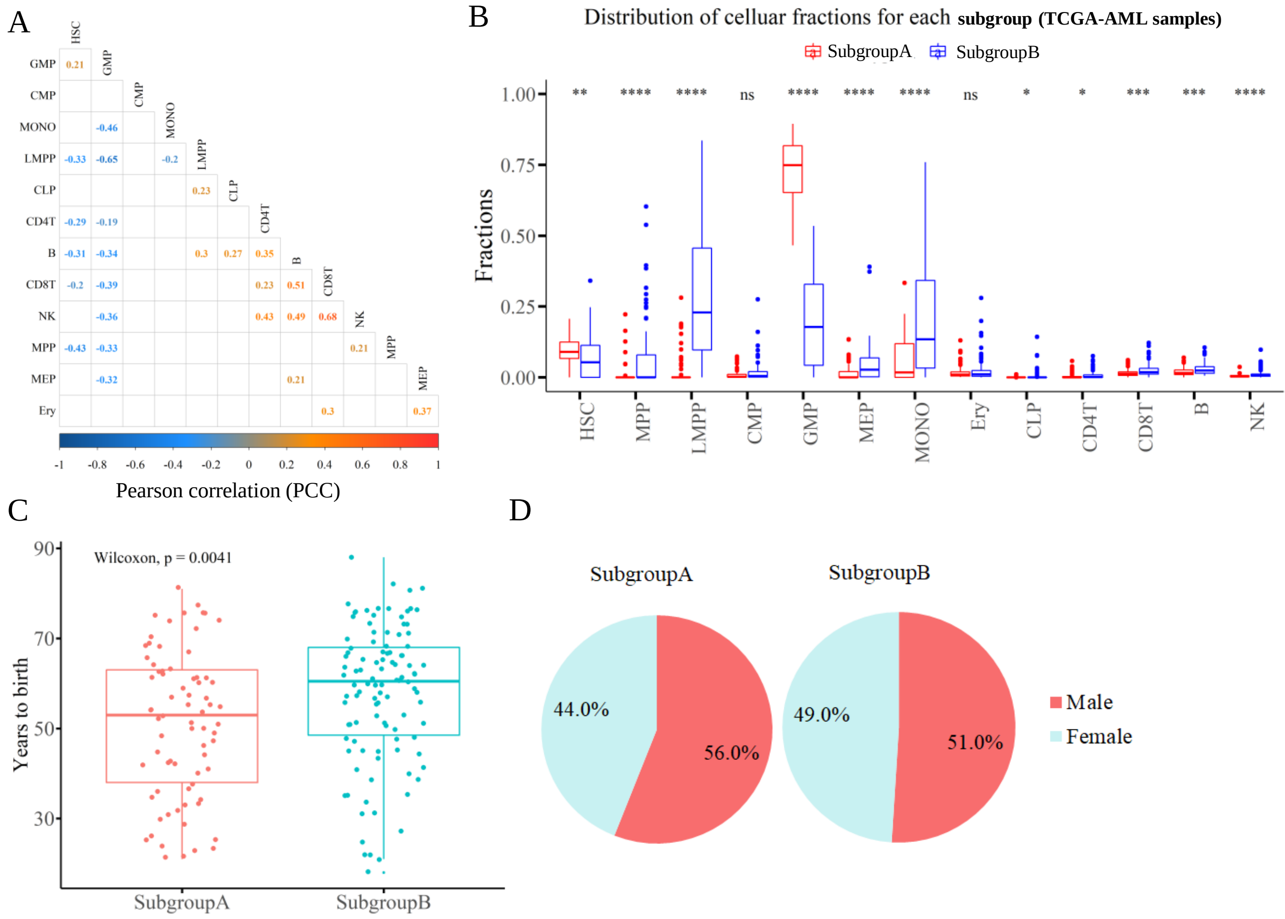
A
Pearson correlation (PCC)
B
Distribution of celluar fractions for each subgroup (TCGA-AML samples)
SubgroupA
SubgroupB
Fractions
HSC
MPP
LMPP
CMP
GMP
MEP
MONO
Ery
CLP
CD4T
CD8T
B
NK
C
Wilcoxon, p = 0.0041
Years to birth
SubgroupA
SubgroupB
D
SubgroupA
SubgroupB
44.0%
56.0%
49.0%
51.0%
Male
Female

A

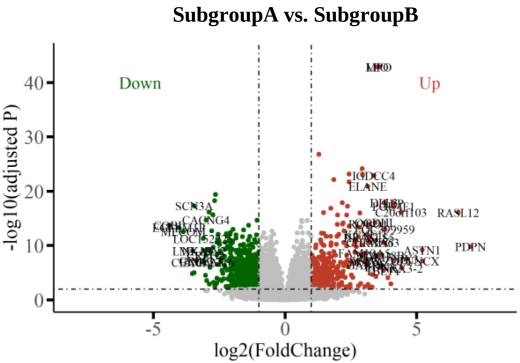

C

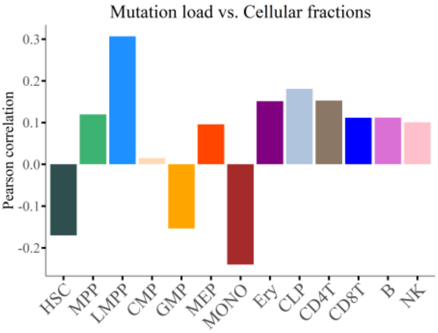

B

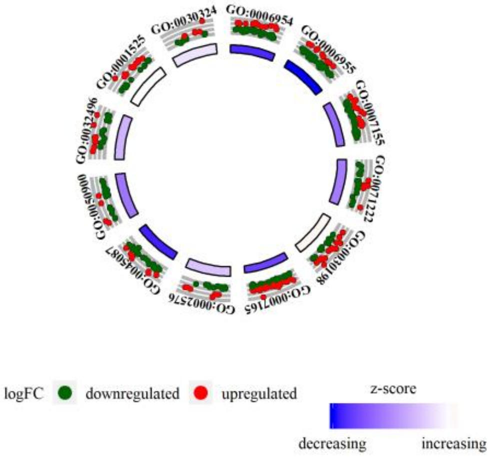

ID	Description
GO:0006954	inflammatory response
GO:0006955	immune response
GO:0007155	cell adhesion
GO:0071222	cellular response to lipopolysaccharide
GO:0030198	extracellular matrix organization
GO:0007165	signal transduction
GO:0002576	platelet degranulation
GO:0045087	innate immune response
GO:0050900	leukocyte migration
GO:0032496	response to lipopolysaccharide
GO:0001525	angiogenesis
GO:0030324	lung development
